# Low-Cost Simulation Kits for Surgical Training in Resource-Limited Settings: A Student-Led Model

**DOI:** 10.7759/cureus.95940

**Published:** 2025-11-02

**Authors:** John Salib, Mark Salib, Matthew Phillips

**Affiliations:** 1 Medicine, St. George's University School of Medicine, St. George's, GRD; 2 General Surgery, Community First Medical Center, Chicago, USA

**Keywords:** low cost devices, low-income resource-limited countries, resource limited setting, simulation kits, simulation models, surgical skills-based training, surgical training model, surgical training simulator

## Abstract

This paper provides a guideline for establishing medical skills training clinics aimed at better preparing future physicians with essential clinical and surgical skills, particularly in underdeveloped regions where high mortality rates are often linked to insufficient hands-on training. In some areas, physicians are forced to perform procedures on live patients without prior practical experience. This study examines simulation kits developed by a student-run surgical organization at a Caribbean medical school and discusses the outcomes of their clinical skills clinics. Results show that systematic learning, deliberate practice, and the use of simulation centers contribute to producing better-trained physicians by offering safe environments for practicing clinical techniques without compromising patient care. Rather than replacing bedside teaching, simulation centers serve as valuable adjuncts that reinforce clinical competence. By highlighting the structure and content of these training clinics, this paper advocates for their adoption, particularly in resource-limited settings, to enhance physician preparedness and support global medical standards while encouraging further research into optimizing such programs.

## Introduction

Global health trends are shifting, with low- and middle-income countries (LMICs) now facing a rapid rise in non-communicable diseases (NCDs) such as heart disease, diabetes, and cancer. This epidemiologic transition is driven by longer life expectancies, urbanization, changing population dynamics, and lifestyle factors such as poor diet, physical inactivity, and tobacco use. The World Health Organization reports that over 77% of all NCD-related deaths now occur in LMICs, countries already struggling with limited resources and the ongoing burden of infectious diseases [1].

One of the biggest obstacles in addressing this growing health crisis is the shortage of trained healthcare professionals. In many African countries, the ratio of physicians to the population remains critically low, often fewer than one doctor per 5,000 people [2]. A lack of specialists further exacerbates the problem, leading to delays in care and poorer health outcomes. Moreover, many healthcare systems in these regions lack the infrastructure for hands-on, skills-based training. While simulation-based education is widely used in high-income settings, it remains scarce in LMICs due to cost and logistical constraints. As a result, many providers are compelled to perform critical procedures for the first time on actual patients, often in high-risk or under-resourced environments. Strikingly, although LMICs house nearly 70% of the world’s population, they account for more than 80% of deaths from surgically treatable conditions [3].

To bridge this widening skills gap, there is a pressing need for innovative, scalable, and context-specific educational solutions. Student-led simulation initiatives represent one such approach, empowering trainees to take an active role in designing, developing, and disseminating training tools tailored to their own learning environments. By harnessing local materials, creativity, and peer collaboration, such models can promote both self-directed learning and sustainability. This approach not only strengthens procedural competence but also cultivates leadership, problem-solving, and resource management skills among future clinicians.

Our intention was to design a low-cost and accessible simulation model that captures the key psychomotor and procedural steps of basic surgical techniques while remaining feasible for students in resource-limited settings. We recognize that our approach differs from traditional or high-fidelity simulation methods; however, our primary goal was to balance educational value with practicality and affordability. This initiative aims to demonstrate that meaningful, hands-on surgical training can be achieved even in settings with limited financial and material resources, ultimately supporting safer surgical practice and more equitable global health outcomes.

## Technical report

Vascular anastomosis

Vascular anastomosis is a vital surgical technique that reconnects or bypasses blood vessels to restore blood flow and circulation. It may involve autologous grafts, such as the saphenous vein or internal mammary artery, or synthetic conduits like polytetrafluoroethylene (PTFE) or Dacron (DuPont, Luxembourg). This technique is commonly used in coronary artery bypass grafting (CABG), vascular trauma repair, and organ transplantation [4].

Indications include vessel damage from trauma, atherosclerosis, aneurysms, and iatrogenic injuries. It is also critical in transplant surgery and for creating vascular access in patients undergoing dialysis. Globally, vascular procedures account for approximately 6% of all surgeries, underscoring their significance in various clinical settings [5]. Despite its utility, vascular anastomosis carries risks, including leakage, thrombosis, hemorrhage, infection, and injury to nearby structures. Anastomotic failure is a significant cause of graft loss and postoperative complications [6]. Proper technique, anticoagulation, and intraoperative imaging are crucial in minimizing these risks.

The materials required for this simulation include a small disposable cup, latex tubing, adhesive or glue, and scissors. Additionally, two needle drivers and forceps are necessary for handling delicate structures. Fine suture material, preferably 4-0 or finer, is recommended to mimic realistic vascular repair. A needle and syringe are also needed to test the integrity of the anastomosis upon completion.

Assembly of the Simulation Kit

**Figure 1:**
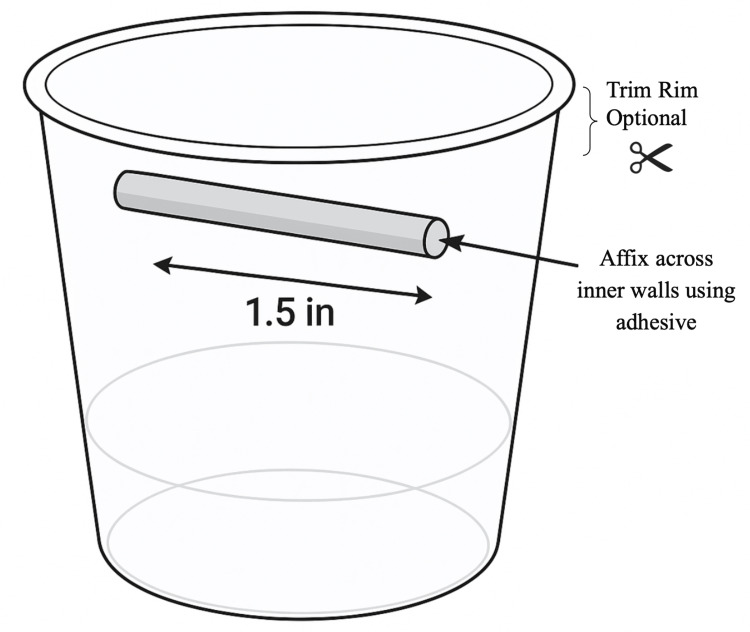
Assembly of a Low-Cost Vascular Anastomosis Simulation Kit. Construction of a vascular anastomosis training model using a disposable cup and latex tubing. A 1.5-inch segment of latex tubing is affixed horizontally to the inner walls of the cup near the rim using adhesive, simulating a vessel for anastomosis practice. The cup’s rim may be trimmed to enhance access and visualization of the operative field. Figure created by the authors using ChatGPT version 4 (OpenAI, San Francisco, California, USA) image generation tool.

Begin by cutting a segment of latex tubing approximately 1.5 inches in length or sized to match the diameter of the disposable cup used. Using adhesive, secure each end of the latex tubing to the opposite inner walls of the cup, ensuring that the tubing remains straight and free of sharp bends. Position the tubing close to the rim of the cup to optimize visualization. If necessary, trim the cup's rim with scissors to improve access and visibility of the simulated surgical field (Figure 1).

Procedure for Vascular Anastomosis

**Figure 2:**
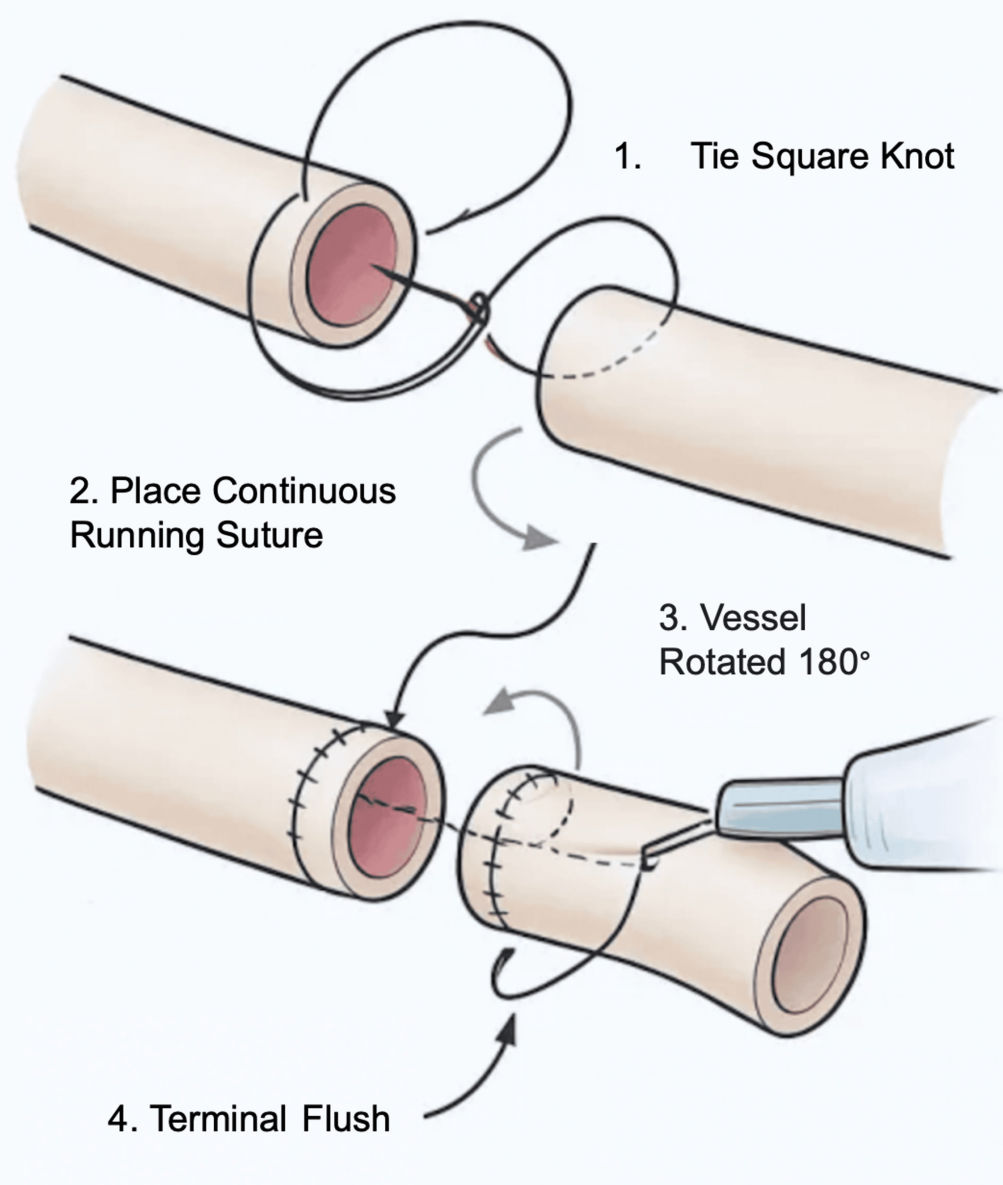
Simulated Vascular Anastomosis Technique Using Low-Cost Training Kit. A continuous vascular anastomosis is performed by suturing from inside to outside at the far end and advancing toward the operator. Perpendicular bites are placed 2 mm from the edge and 1 mm apart. After completing the posterior wall, the vessel is rotated to suture the anterior wall. The lumen is flushed for patency, and the anastomosis is secured with a final knot. Figure created by the authors using ChatGPT version 4 (OpenAI, San Francisco, California, USA) image generation tool.

The procedure for vascular anastomosis begins by suturing in a direction toward the operator to maximize depth perception and visibility. Start by inserting the needle from the inside of the vessel to the outside at the far end, and tie a square knot on the external surface to avoid intraluminal obstruction. With the two vessel ends aligned, perform a continuous suture using perpendicular bites approximately 2 mm from the vessel edge and spaced 1 mm apart, advancing the suture line toward yourself. After completing the posterior wall, rotate or reposition the vessel and repeat the same technique to close the anterior wall. Finally, tie off the suture with a secure knot to complete the anastomosis. Once complete, flush the vessel with water using a needle and syringe to test the anastomotic linkage for leaks (Figure 2).

Burn debridement

Surgical debridement is a procedure used to remove non-viable tissue from a wound, most commonly in the context of burns, ulcers, or necrotic injuries. It may be performed under local or general anesthesia, depending on the size, depth, and location of the affected area. The primary goal is to expose viable tissue to facilitate healing and prepare the wound bed for further interventions such as skin grafting or advanced wound care [7]. Surgical debridement is typically indicated in cases where necrotic tissue, gangrene, or infection is present and where the body's natural healing response is insufficient [8]. Removing devitalized tissue not only promotes faster healing but also reduces the risk of bacterial colonization and systemic infection. Although surgical debridement is effective and often necessary, it carries potential risks, including bleeding, infection, pain, and inadvertent removal of healthy tissue [9,10]. Other forms of debridement, such as enzymatic, biological, or mechanical, may be considered depending on the wound type and clinical setting, although this article focuses specifically on surgical methods [11].

The materials needed for this simulation include a manikin, box, or container to represent the patient, along with saran wrap and a blow dryer to simulate skin and blistering effects. A needle and syringe filled with water are used to replicate fluid-filled blisters. Forceps and scissors are required for wound manipulation and debridement. Hand cream serves to simulate wound moisture, and a sterile dressing is applied as the final step in the wound care process.

Assembling the Simulation Kit

**Figure 3:**
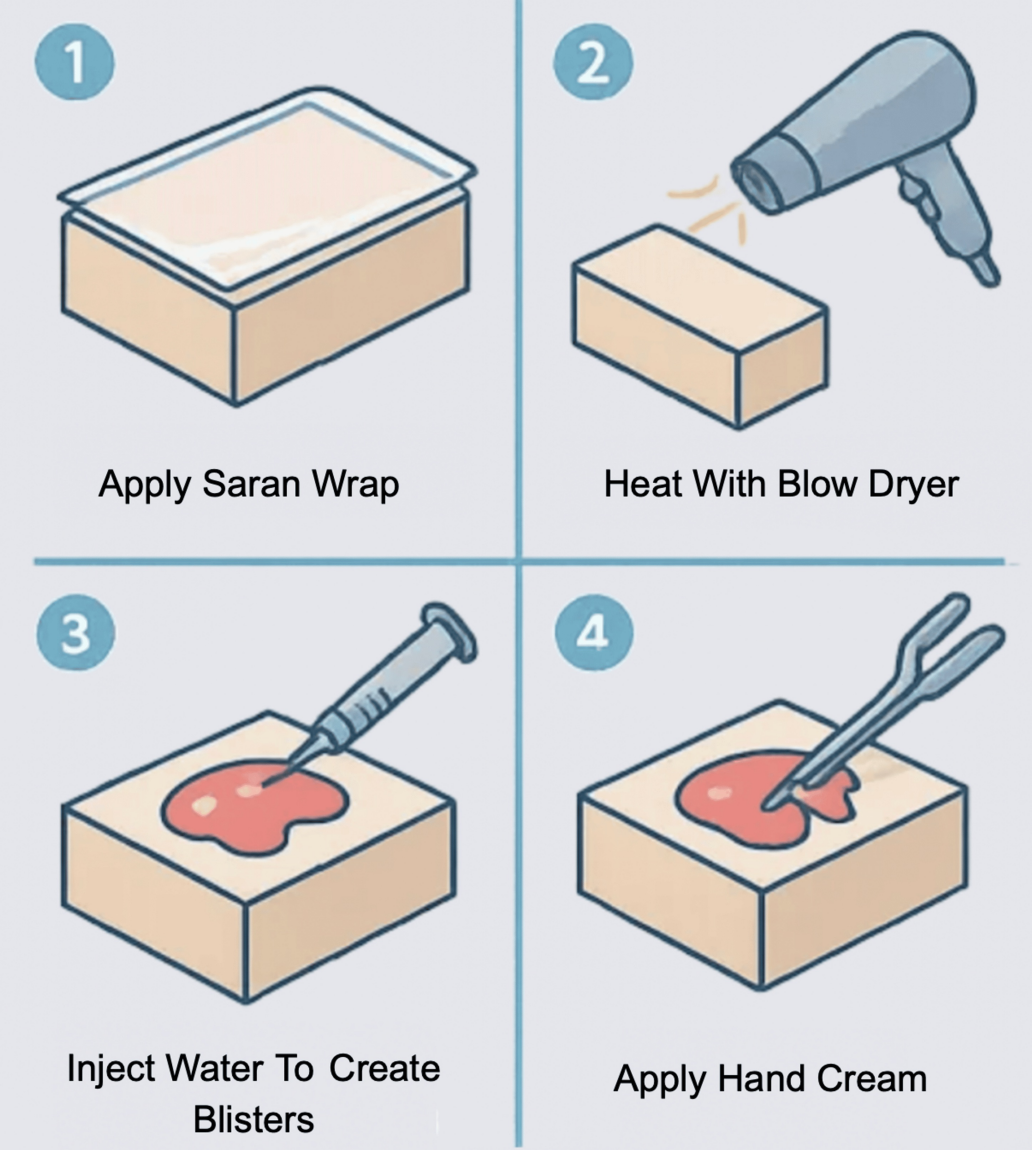
Assembly of a Low-Cost Burn Wound Simulation Model. Simulation model for burn wounds using household materials. Saran wrap is crumpled and heated with a blow dryer over a disinfected surface to mimic blistered skin. Water is injected beneath the wrap to simulate fluid-filled blisters, and hand cream is applied for a moist appearance. The model is suitable for practicing burn wound assessment, debridement, and dressing techniques. Figure created by the authors using ChatGPT version 4 (OpenAI, San Francisco, California, USA) image generation tool.

Begin by cleaning and disinfecting the surface of the object that will be used as the simulated patient. Apply or crumple saran wrap over the surface to replicate the irregular texture of damaged skin. Using a blow dryer, gently heat the saran wrap so that it adheres closely to the surface, creating the appearance of tightened, blistered skin. To simulate fluid-filled blisters, use a needle and syringe to carefully inject small amounts of water into pockets of air beneath the saran wrap. This helps mimic the appearance and behavior of burn-induced blistering. Hand cream may be applied to give the surface a moist, exudative appearance. Once complete, sterile dressings can be used to practice dressing techniques (Figure 3).

Procedure for Burn Debridement

**Figure 4:**
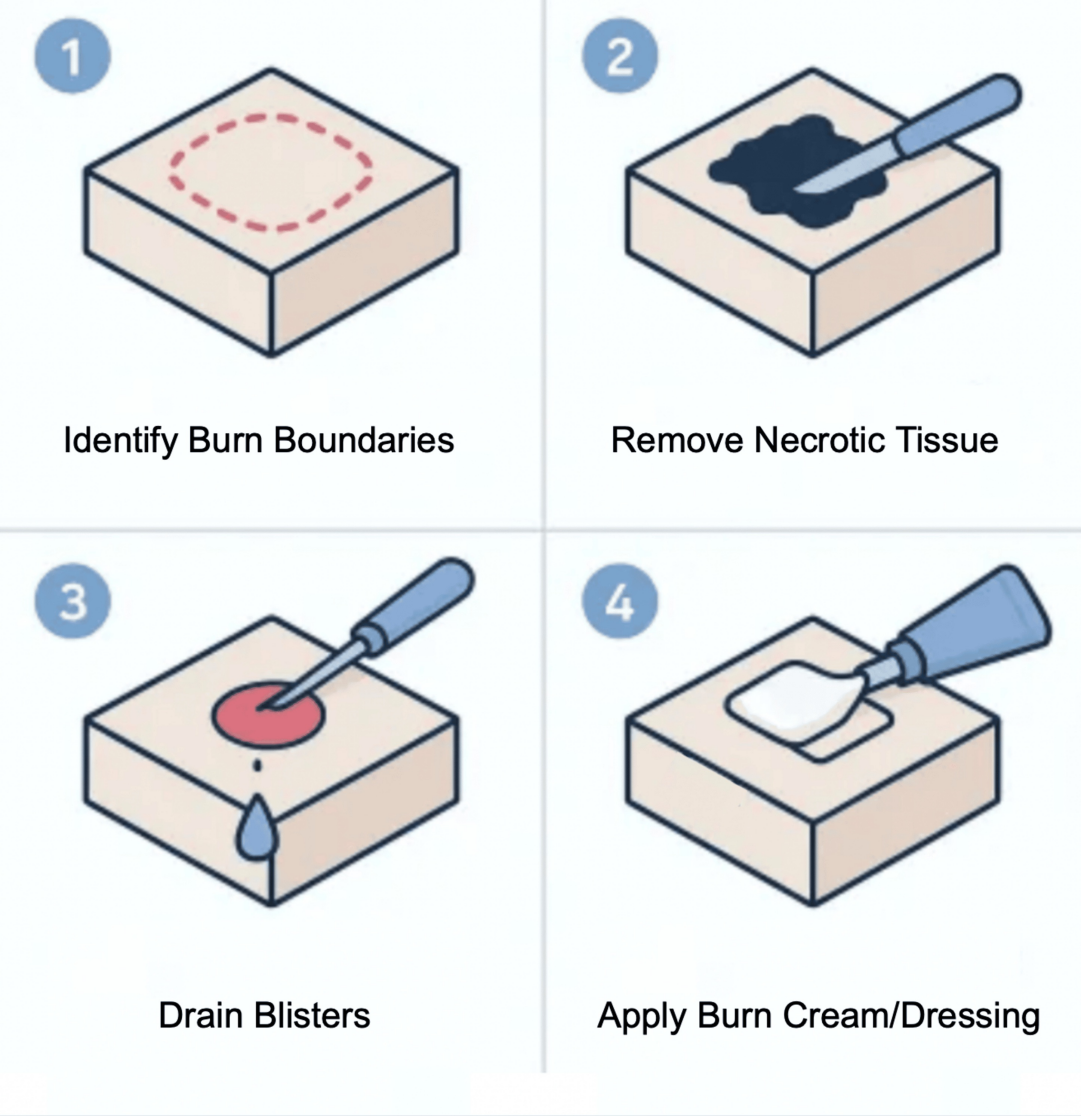
Simulated Burn Debridement Procedure Using Low-Cost Model. Stepwise simulation of burn wound debridement. The burn area is first outlined, followed by careful removal of simulated necrotic tissue using scissors and forceps. Fluid-filled blisters are then drained, and burn cream is applied to the exposed surface. The wound is finished with placement of a sterile dressing to replicate post-debridement care. Figure created by the authors using ChatGPT version 4 (OpenAI, San Francisco, California, USA) image generation tool.

This simulation is designed to help practice the technique of surgical debridement in the context of a severe burn injury. Begin by identifying and outlining the boundaries of the simulated burn area. Using forceps and scissors, carefully remove the burn debris, mimicking the removal of necrotic tissue. In clinical settings, this step is typically painful for patients and requires a gentle, deliberate technique; approach the simulation with the same care. Debridement should continue until all non-viable material is removed, staying within the marked boundaries of the burn. Once complete, simulate the drainage of blisters by gently releasing fluid from bubble-like areas created earlier. After debridement, apply a layer of burn cream to the exposed tissue and cover the area with a sterile dressing to simulate appropriate wound care (Figure 4).

Lipoma excision

A lipoma is a slow-growing, benign mass of adipose tissue typically located between the skin and the underlying muscle, often lying just beneath the fascial layer [12]. These soft, mobile lesions are usually asymptomatic and most commonly occur on the upper back, shoulders, arms, and thighs. While lipomas are not malignant, they may be removed for cosmetic reasons or when they cause discomfort due to impingement on adjacent structures. Treatment options include surgical excision or liposuction, particularly for larger lesions [13].

Excision involves careful dissection of the lipoma from surrounding tissues using a scalpel or scissors, generally performed under local anesthesia. Although often considered a minor procedure, the degree of invasiveness depends on the lesion’s size, depth, and anatomical location. Lipomas do not possess a true capsule; instead, they are surrounded by compressed fibrous connective tissue that may give the appearance of a capsule during dissection. In some cases, lipomas may form close attachments to adjacent neurovascular structures, increasing the risk of injury during excision. Despite the generally low-risk profile, potential complications include infection, hematoma formation, damage to nearby structures, and postoperative contour deformities [14].

When replicating this procedure using simulation kits, certain anatomical limitations are inevitable. Simulated tissues may not fully reproduce the fibrofatty texture or pseudo-capsular layer of a true lipoma, and the absence of adjacent neurovascular structures limits opportunities to practice safe dissection planes. Nonetheless, such models provide valuable opportunities for learners to practice incision planning, blunt and sharp dissection, tissue handling, and wound closure in a controlled environment.

The materials required for this simulation include a dish sponge, preferably one with a rough-textured side to represent the tissue base. Condoms are used to encase the simulated tumor contents, which can be created using Jell-O or cornstarch to approximate the consistency of adipose tissue. Additional tools include glue for assembly, forceps, a needle driver, scissors, suture, and a scalpel for dissection and closure. A marker is used to outline the incision site, and a needle with a syringe may be utilized for fluid injection or to test the setup.

Assembling the Simulation Kit

**Figure 5:**
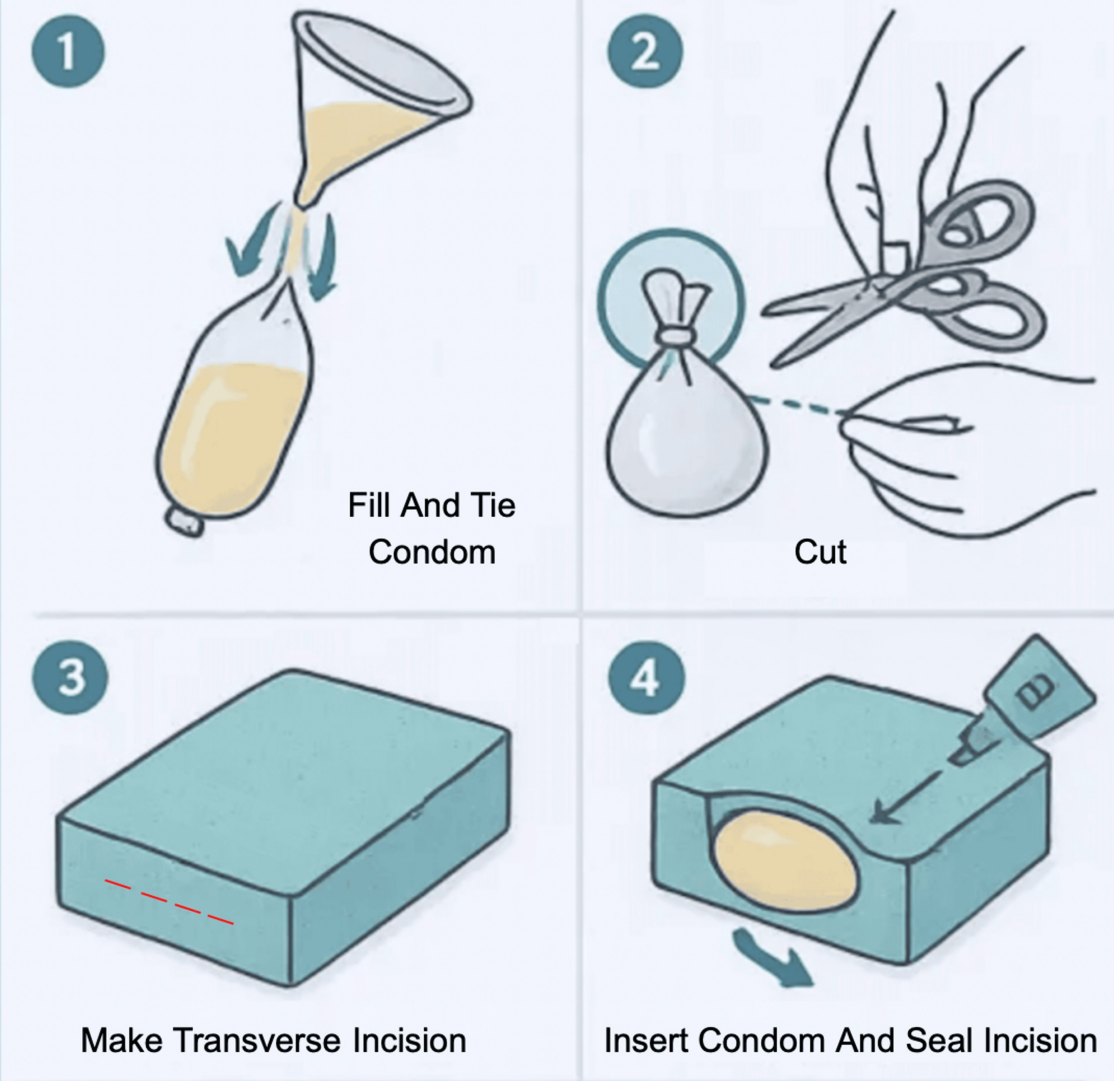
Assembly of a Benign Tumor Dissection Simulation Model. A simulated benign tumor is created by filling a condom with a thick Jell-O or cornstarch solution and embedding it into a dish sponge via a lateral incision. The mass is secured with glue to mimic realistic texture and positioning. This model allows for practice in soft tissue dissection, excision, and basic surgical techniques. Figure created by the authors using ChatGPT version 4 (OpenAI, San Francisco, California, USA) image generation tool.

Begin by preparing a thick Jell-O or cornstarch solution to mimic the internal consistency of a benign tumor. Carefully pour the solution into a condom and tie the open end, trimming off any excess material to form a smooth, round structure with well-demarcated borders. This not only provides a realistic texture but also facilitates easier dissection during the simulation. Using a scalpel, make a small lateral incision, approximately 1-2 cm from the base, on the side of the dish sponge. Apply glue to the surface of the filled condom and insert it into the sponge so that it rests centrally at the base. Once positioned, apply additional glue to seal the entry incision, ensuring that the simulated mass remains securely embedded within the sponge. The resulting model can then be used to simulate soft tissue dissection, excision, and basic surgical techniques for the removal of benign tumors (Figure 5).

Procedure for Lipoma Excision

**Figure 6:**
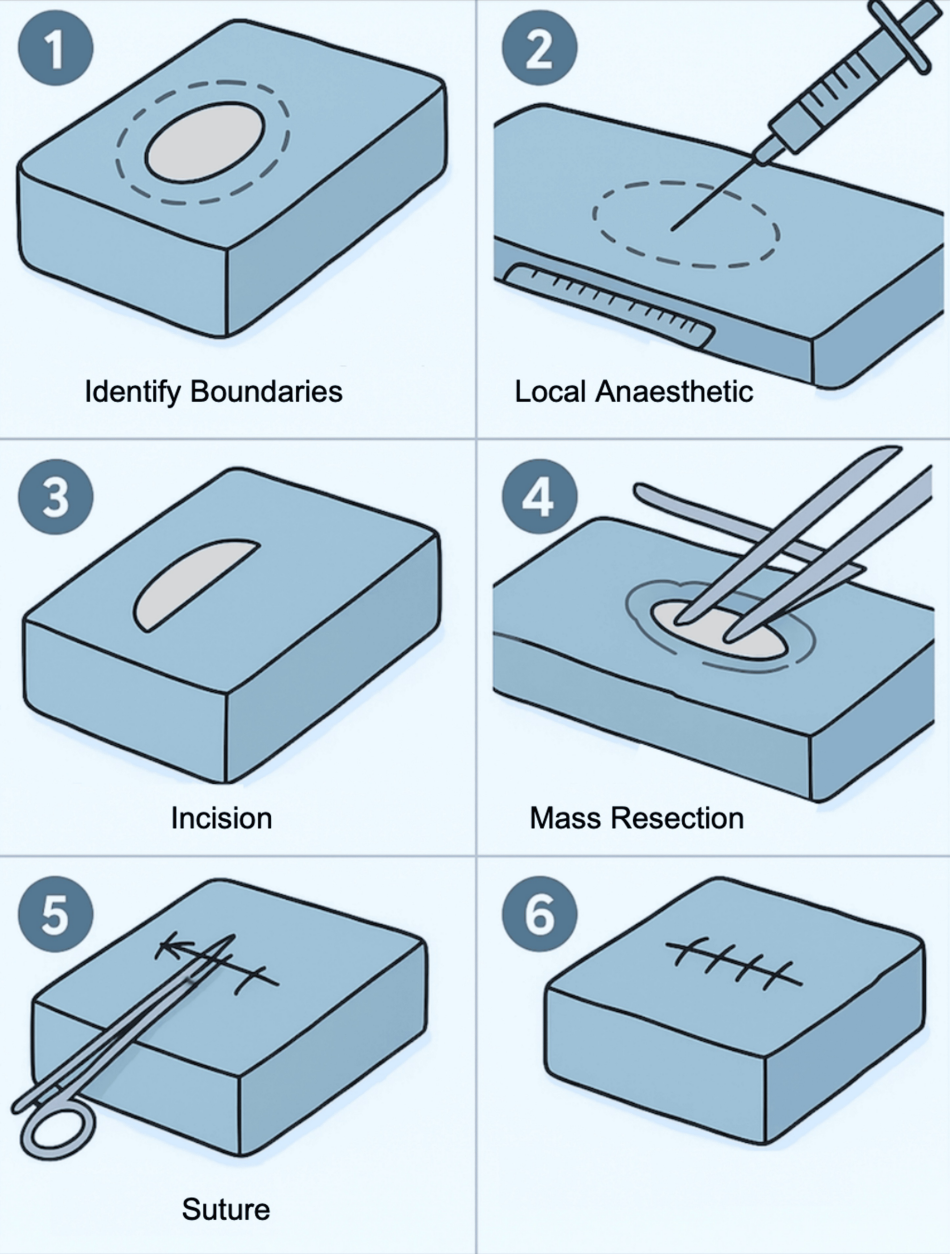
Simulated Lipoma Excision Procedure Using Embedded Tumor Model. Step-by-step simulation of lipoma excision using a sponge-embedded mass. The procedure includes marking the lesion, administering simulated local anesthesia, making a longitudinal incision, and carefully dissecting around the mass to preserve its integrity. The lipoma is excised intact, followed by wound cleaning and closure with sutures to simulate post-excision care. Figure created by the authors using ChatGPT version 4 (OpenAI, San Francisco, California, USA) image generation tool.

This simulation is designed to mimic the excision of a superficial lipoma while maintaining the integrity of the embedded tumor (a condom filled with Jell-O or cornstarch). Begin by marking the boundaries of the simulated lipoma on the sponge surface using a marker, and administer simulated local anesthesia to the site using a needle and syringe. Make a longitudinal incision directly over the mass with a scalpel, extending through the simulated skin layer to the pseudo-capsular plane. Using gentle blunt dissection, incise the pseudo-capsule and apply digital pressure or forceps to express the lipomatous mass through the incision. This technique minimizes unnecessary circumferential dissection and reduces the risk of injury to nearby neurovascular structures, which may lie in close contact with the lipoma in vivo. Once the mass is delivered intact, inspect the wound bed to ensure complete removal and absence of residual adipose tissue. Clean the simulated surgical field, then close the incision using the appropriate suturing technique. This approach emphasizes controlled tissue handling, anatomical awareness, and safe procedural sequencing, all critical skills in minor surgical procedures performed in resource-limited settings (Figure 6).

Cricothyrotomy

A cricothyrotomy is an emergency surgical airway procedure performed when conventional endotracheal intubation is impossible or ineffective, such as in cases of severe oral or maxillofacial trauma, uncontrolled oral bleeding, massive vomiting, or anatomical abnormalities obstructing the airway [15]. Due to its high-risk nature, cricothyrotomy should only be attempted by providers with extensive training and experience and solely in life-threatening situations where ventilation cannot be otherwise secured [16]. The procedure is contraindicated if anatomical landmarks are not identifiable, in the presence of active tracheal or laryngeal infections, or in pediatric patients, for whom less invasive airway management techniques are preferred [17]. Potential complications include esophageal or tracheal injury, thyroid gland perforation, hypercapnia from inadequate ventilation, and aspiration of blood, all of which can significantly worsen patient outcomes [16].

The materials needed for this simulation include thin latex tubing and textured rigid plastic tubing to replicate tracheal structures. A scalpel is required for performing the incision, while a tracheal tube or cricothyrotomy tube is used for airway insertion. Suture, a stabilizing band, and a guidewire are essential for securing the airway and guiding placement. Antiseptic solution is included to simulate procedural preparation. Hot glue or duct tape is used for model assembly, and a ball pump can be incorporated to mimic ventilation or airway inflation.

Assembling the Simulation Kit

**Figure 7:**
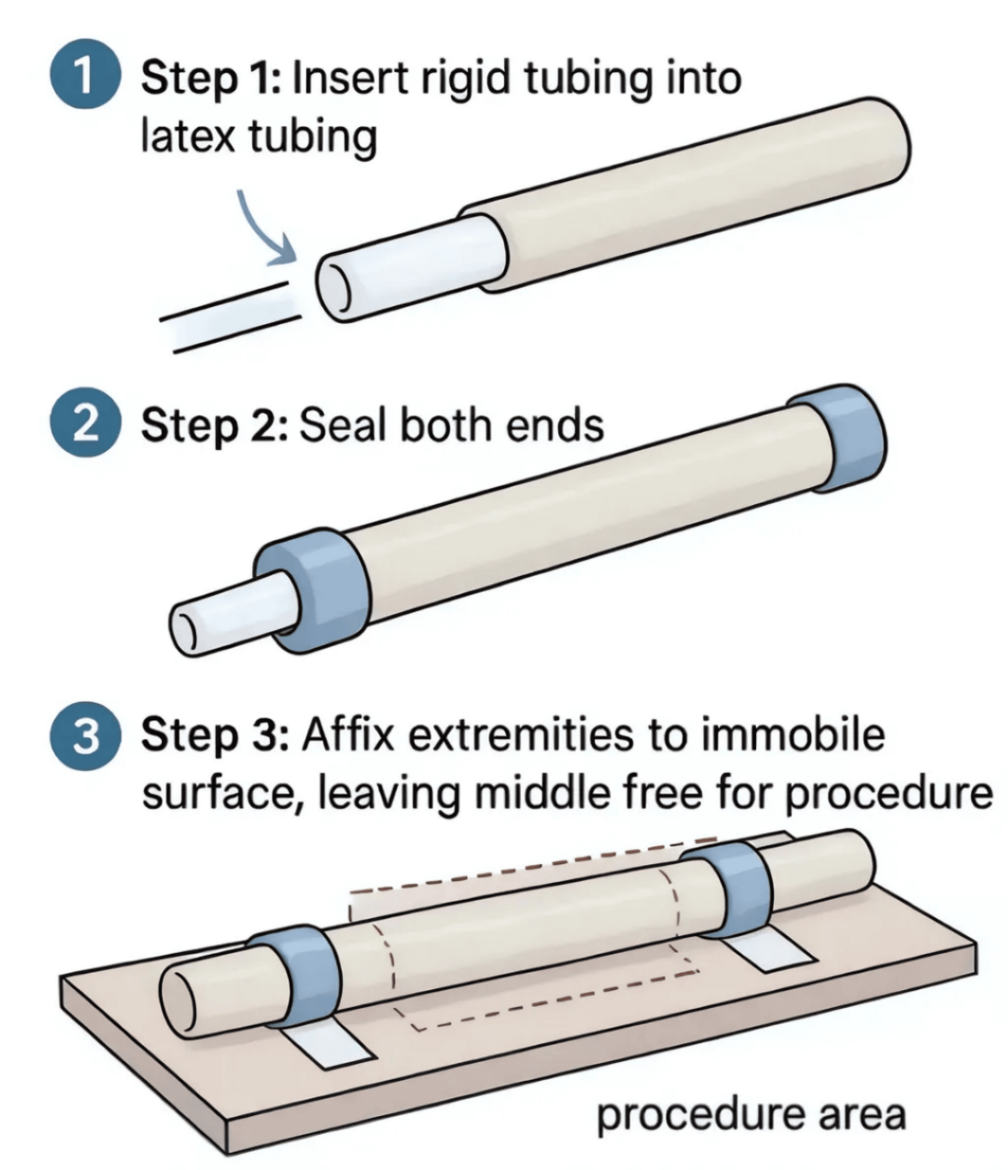
Assembly of a Low-Cost Airway Simulation Model. An airway model is constructed by inserting rigid plastic tubing into thin latex tubing to mimic tracheal structure. The ends are sealed with duct tape or hot glue, and the model is secured to a stable surface. This setup facilitates practice of airway access procedures such as cricothyrotomy and tracheal intubation. Figure created by the authors using ChatGPT version 4 (OpenAI, San Francisco, California, USA) image generation tool.

Begin by inserting the textured rigid plastic tubing inside the thin latex tubing to simulate the airway structure. Securely seal both ends of the assembled tubing using duct tape or hot glue to prevent slippage and maintain structural integrity. Affix the model firmly to an immobile surface, such as a table, to provide stability during the simulation. This setup allows for realistic practice of airway access techniques, including cricothyrotomy and tracheal intubation (Figure 7).

Procedure for Cricothyrotomy

**Figure 8:**
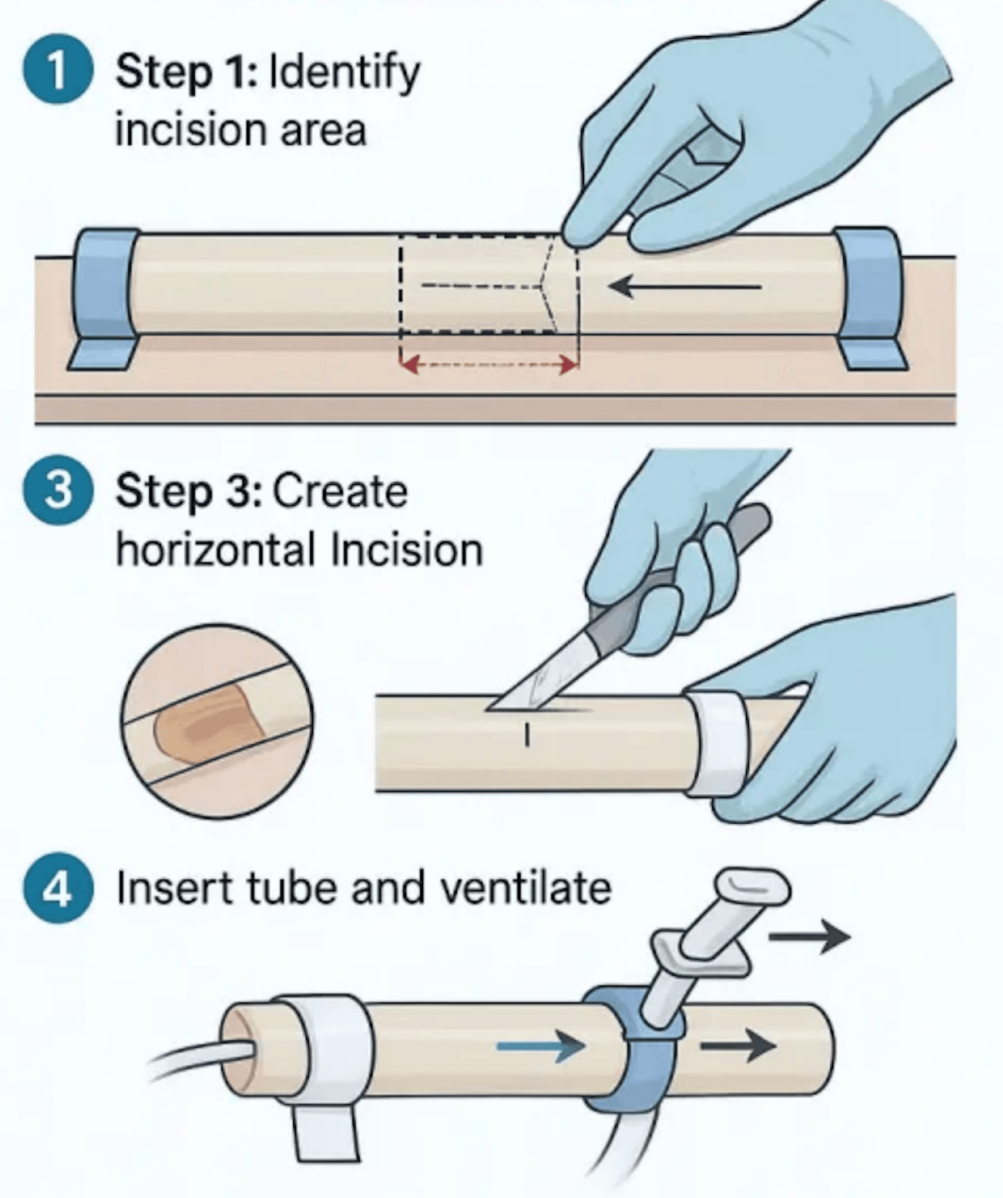
Simulated Cricothyrotomy Procedure Using Airway Model. Step-by-step simulation of emergency airway access. The procedure includes antiseptic preparation, vertical and horizontal incisions through the cricothyroid membrane, insertion and inflation of the airway tube, securing the tube, and initiating ventilation to restore airway patency. Figure created by the authors using ChatGPT version 4 (OpenAI, San Francisco, California, USA) image generation tool.

The goal of this procedure is to establish an emergency airway through the cricothyroid membrane. Although anatomical landmarks such as the cricoid and thyroid cartilages can be challenging to replicate in simulation, the exercise focuses on restoring airway patency without relying on palpation. Begin by disinfecting the simulated surgical area with antiseptic. Using a scalpel, make a vertical incision from the inferior border of the thyroid cartilage down to the cricoid cartilage. Next, create a horizontal incision through the cricothyroid membrane and insert the cricothyrotomy or tracheal tube. Inflate the tube cuff and remove the guidewire. Secure the tube in place using sutures or a stabilizing band to prevent displacement. Finally, initiate ventilation through the tube using either a ball pump or the standard mouth-to-tube technique (Figure 8).

## Discussion

The primary goal of medical education is to train clinically competent physicians through a combination of foundational sciences and hands-on experience. While most medical schools emphasize basic science instruction in the early years, student-led initiatives often provide vital opportunities for early clinical exposure. Traditionally, bedside teaching has been the cornerstone of clinical training, enabling students to refine diagnostic and procedural skills through direct patient care. However, factors such as limited patient access, ethical restrictions, and time constraints have increasingly restricted these opportunities, resulting in greater reliance on simulation-based learning. Simulation environments help bridge this educational gap by allowing trainees to develop procedural proficiency in a safe and structured setting, improving confidence and patient safety [17,18].

Structured simulation training is essential in low-resource settings. In many regions with limited infrastructure, including parts of Africa and the Caribbean, surgical trainees often perform procedures on live patients with minimal hands-on experience due to faculty supervision and training materials shortages. This contributes to higher complication rates from surgically treatable conditions [19,20]. Low-cost simulation models, therefore, represent not only an educational innovation but also a potential public health intervention, providing scalable, reproducible tools for safe skill development.

Pre- and post-clinic survey data were analyzed using McNemar’s test for paired nominal variables to assess the significance of observed changes in student confidence and perceived procedural readiness. This test was selected because it evaluates changes in dichotomous (yes/no) responses from the same participants before and after an intervention, accounting for within-subject variability. Specifically, the proportion of students who initially reported low confidence but later indicated increased confidence following simulation training (i.e., discordant pairs) was compared to those whose responses did not change. Among the 211 participants, 141 students who initially reported lacking confidence before the clinic expressed increased confidence after completing the session, while only nine participants showed no improvement or a decline in confidence. The resulting chi-square statistic was 51.4 (1 df), corresponding to p < 0.001, indicating that the observed improvements were highly statistically significant and unlikely to have occurred by chance. Similar results were obtained for perceived procedural readiness, where 132 students reported increased preparedness after the clinic compared to 11 who did not demonstrate change (χ² = 47.8, p < 0.001). These findings quantitatively confirm that the gains in self-reported confidence and readiness observed in this study represent meaningful educational effects of simulation-based training rather than random variation.

To evaluate expert perception of the simulation models, 12 surgeons (attending surgeons and recent graduates) completed a structured post-workshop survey using a five-point Likert scale (1 = strongly disagree to 5 = strongly agree). The survey assessed four domains: anatomical realism, educational suitability, procedural accuracy, and training applicability. Overall, 75% (9/12) of respondents rated the models as “well-suited” (≥4) for teaching basic surgical techniques and instrument handling in a student-led setting. The mean Likert score across all domains was 4.3 ± 0.6, indicating strong agreement regarding the models’ educational utility. However, 33% (4/12) of respondents provided lower ratings (≤3) for anatomical realism, citing limitations in the replication of tissue planes, fascia, and neurovascular structures. Despite these anatomical constraints, experts emphasized that the models effectively demonstrated the procedural flow and decision-making process required in basic surgical practice.

**Figure 9:**
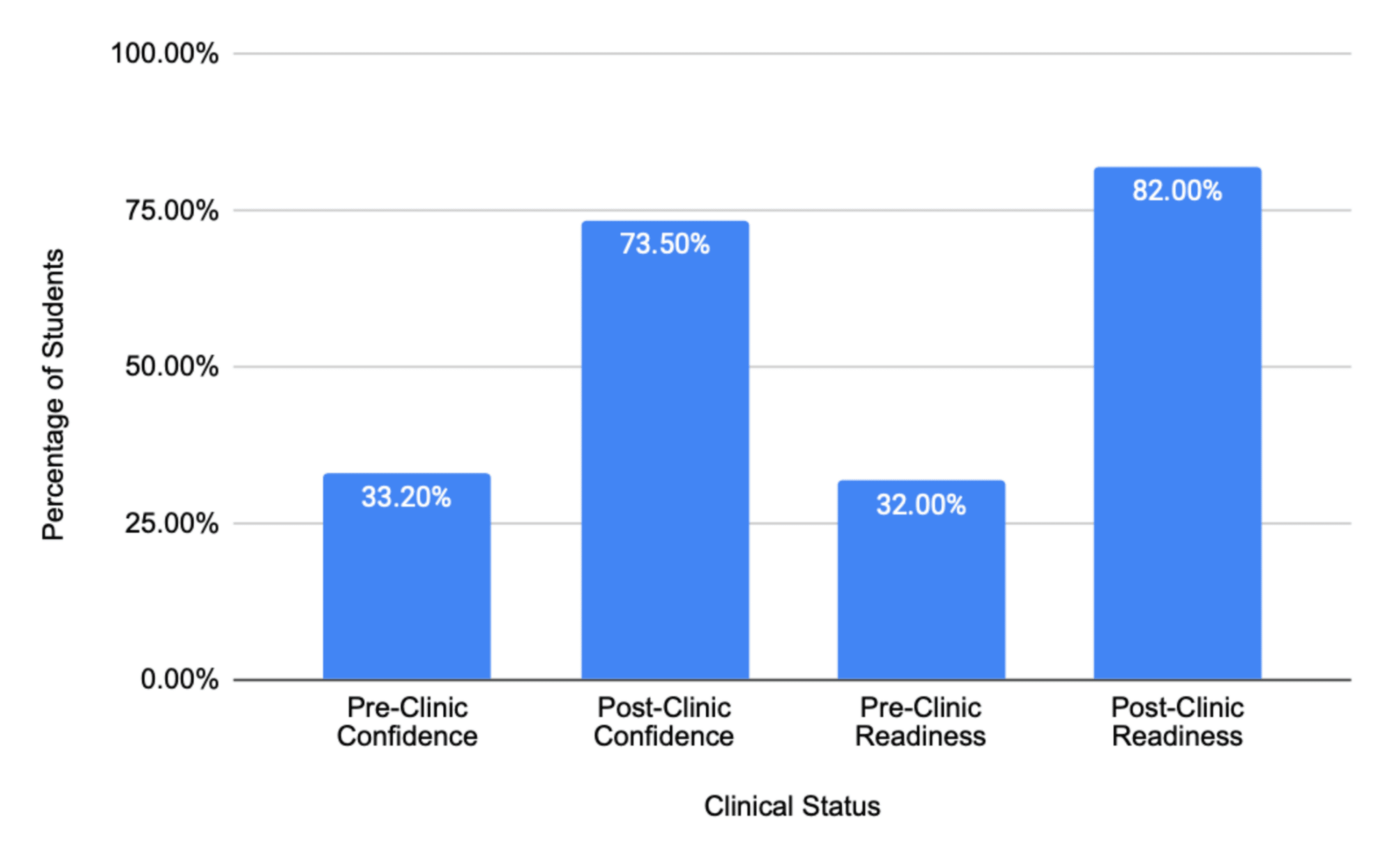
Improvement in Student Confidence and Readiness Following Simulation Clinics. Bar chart showing self-reported confidence and perceived readiness of medical students before and after participating in low-cost, simulation-based clinical skills training. Confidence rose from 33.2% to 73.5%, while readiness to perform procedures on real patients increased from 32% to 82%. These results highlight the effectiveness of structured, hands-on simulation in improving clinical self-efficacy and procedural preparedness among trainees.

These findings underscore that functional fidelity, the model’s ability to teach the key psychomotor and procedural steps, may be more important than perfect anatomical accuracy in early surgical education. Future iterations of these kits could benefit from expert-guided refinements to better simulate tactile feedback and anatomical detail. In addition, while the immediate post-clinic survey demonstrated significant gains in student confidence (p < 0.001, McNemar’s test), future studies should incorporate long-term follow-up assessments (e.g., four to eight weeks post-intervention) to evaluate knowledge retention and sustained skill acquisition. Tracking student performance in subsequent clinical rotations or Objective Structured Clinical Examinations (OSCEs) could further validate the lasting educational impact of this low-cost, simulation-based approach. All values are summarized in Figure 9.

These findings demonstrate that even brief, low-cost simulation sessions can substantially increase medical students' confidence and procedural preparedness. These sessions effectively bridge the gap between theoretical instruction and real-world application by providing a structured, low-risk environment for repetitive practice and immediate feedback. This benefit is particularly pronounced in resource-limited settings, where opportunities for supervised procedural training on live patients are often scarce.

In Miami, Florida, a team of 12 surgical professionals comprising experienced surgeons and recent medical graduates evaluated the educational value of the simulation models. Using a structured post-clinic survey, 75% (9/12) of respondents rated the models as “well-suited” for teaching surgical techniques and instrument handling in early-stage trainees. Experts highlighted that the kits effectively demonstrated the procedural sequence and decision-making required for basic surgical skills while fostering a safe, low-pressure learning environment. A minority of respondents (33%, 4/12) noted that while the models were appropriate for introductory training, their limited anatomical realism would make them less suitable for advanced learners or high-resource settings where high-fidelity simulators are available. Nonetheless, the consensus supported the kits as practical, scalable, and educationally valuable tools, particularly in medical schools and hospitals constrained by cost or infrastructure.

However, these low-cost simulation kits are not without limitations. Their ability to replicate the complete surgical environment remains restricted, as elements such as real-time complications, procedural urgency, and intraoperative stress cannot be fully simulated. Anatomical fidelity also poses challenges. For instance, vascular models provide exposure fields that are broader than in clinical practice and use tubing that is more rigid than natural vessels. Burn models accurately replicate only second-degree burns and fail to reproduce the texture of necrotic tissue, while lipoma models lack the fragmented and irregular consistency of true adipose lesions. Similarly, cricothyrotomy models omit key anatomical landmarks and spatial relationships in the human neck.

Despite these shortcomings, the educational impact of these kits is clear. Their accessibility, ease of assembly, and improved learner outcomes underscore their value as sustainable teaching tools. When integrated into a structured, feedback-oriented curriculum, such low-cost simulations can enhance procedural readiness, reduce learner anxiety, and promote safer, more competent patient care, particularly in regions where traditional simulation resources remain limited.

**Table 1:** Strengths and Clinical Limitations of Low-Cost Simulation Kits. A comparative overview of four simulation models used in student-led surgical training clinics. While each kit supports procedural learning through accessible, reusable materials, they also exhibit notable clinical limitations. For example, vascular and cricothyrotomy kits effectively teach core techniques but lack anatomical realism. Burn and lipoma models enable basic practice but do not replicate the complexity or texture of real tissue. Understanding these trade-offs can inform future improvements and guide appropriate use in early clinical education.

Kit Type	Strengths	Clinical Limitations
Vascular access	Clear vessel visualization; reusable tubing allows repeated practice	Rigid tubing lacks compressibility; exposure field wider than in real anatomy
Burn management	Simulates blistering/sloughing well; good for basic debridement steps	Limited to second-degree burns; doesn’t capture necrotic texture or dry wound beds
Lipoma excision	Cohesive structure supports stepwise dissection; good for scalpel technique	Unrealistic consistency; lacks fragmentation or deterioration common in real lipomas
Cricothyrotomy	Supports learning key airway entry steps; safe for repetition	Inaccurate anatomical landmarks; spacing and depth don’t mimic real neck anatomy

This study demonstrates that low-cost, student-led simulation clinics can significantly enhance medical students’ confidence, procedural competence, and understanding of clinical relevance. Despite being constructed from inexpensive, readily available materials, the kits were widely recognized by both learners and experienced clinicians as practical and effective educational tools. The substantial gains in confidence and self-reported procedural readiness highlight the value of incorporating simulation-based training early in medical education. These findings support the broader adoption of scalable, hands-on training models to bridge the gap between theoretical learning and clinical proficiency, particularly in resource-limited institutions where traditional clinical exposure opportunities are constrained.

## Conclusions

This study demonstrates that low-cost, student-led simulation clinics can be powerful tools for improving procedural education in resource-limited environments. Using simple, readily available materials, we successfully created four surgical training kits, including vascular anastomosis, burn debridement, lipoma excision, and cricothyrotomy, that captured each procedure's key psychomotor and technical components. The results revealed significant improvements in students’ confidence and perceived procedural readiness, as confirmed by McNemar’s test showing highly significant gains (p < 0.001). Moreover, despite their limited anatomical fidelity, expert evaluations affirmed that the models were educationally effective, functionally realistic, and well-suited for teaching fundamental surgical techniques. These findings highlight that meaningful surgical skill development does not require expensive, high-fidelity simulators; effective learning can be achieved through accessible, well-structured, and reproducible models. Integrating such low-cost simulation initiatives into early medical education can bridge the gap between theoretical instruction and clinical practice, fostering greater competence, confidence, and patient safety. Ultimately, this approach offers a sustainable and scalable framework to strengthen surgical training and address the growing procedural skills gap faced by medical trainees worldwide.
